# Reducing-Agent-Free Instant Synthesis of Carbon-Supported Pd Catalysts in a Green Leidenfrost Droplet Reactor and Catalytic Activity in Formic Acid Dehydrogenation

**DOI:** 10.1038/srep26474

**Published:** 2016-05-20

**Authors:** Dong-Wook Lee, Min-Ho Jin, Young-Joo Lee, Ju-Hyoung Park, Chun-Boo Lee, Jong-Soo Park

**Affiliations:** 1 Advanced Materials and Devices Laboratory, Korea Institute of Energy Research (KIER), 152 Gajeongro, Yuseong, Daejeon 305-343, Republic of Korea; 2 Clean Fuel Laboratory, Korea Institute of Energy Research (KIER), 152 Gajeongro, Yuseong, Daejeon 305-343, Republic of Korea

## Abstract

The development of green synthesis methods for supported noble metal catalysts remains important challenges to improve their sustainability. Here we first synthesized carbon-supported Pd catalysts in a green Leidenfrost droplet reactor without reducing agents, high-temperature calcination and reduction procedures. When the aqueous solution containing Pd nitrate precursor, carbon support, and water is dripped on a hot plate, vapor layer is formed between a solution droplet and hot surface, which allow the solution droplet to be levitated on the hot surface (Leidenfrost phenomena). Subsequently, Pd nanoparticles can be prepared without reducing agents in a weakly basic droplet reactor created by the Leidenfrost phenomena, and then the as-prepared Pd nanoparticles are loaded on carbon supports during boiling down the droplet on hot surface. Compared to conventional incipient wetness and chemical synthetic methods, the Leidenfrost droplet reactor does not need energy-consuming, time-consuming, and environmentally unfriendly procedures, which leads to much shorter synthesis time, lower carbon dioxide emission, and more ecofriendly process in comparison with conventional synthesis methods. Moreover, the catalysts synthesized in the Leidenfrost droplet reactor provided much better catalytic activity for room-temperature formic acid decomposition than those prepared by the incipient wetness method.

For synthesis of metal nanoparticles, metal precursors are generally reduced through thermal decomposition at high temperature^1^, electrochemical routes^2^,^3^,^4^,^5^ and chemical pathways^6^,^7^,^8^,^9^,^10^,^11^,^12^,^13^,^14^ using various reducing agents such as sodium borohydride, hydrazine, ethylene glycol, ascorbic acid etc. However, most of the strong reducing agents are toxic and may cause injury to human organs^15^, and electrochemical routes require additional electrochemical machines. Thus, the simple and ecofriendly reduction routes of metal precursors are highly desired for the improvement in productivity and sustainability of synthetic processes of metal-based nanomaterials^15^. For example, many of recently reported processes used natural and renewable sources as a reducing agent for metal nanoparticle synthesis^16^, such as plant extracts, biopolymers, vitamins, protein, peptides, and sugars^17^,^18^,^19^,^20^,^21^,^22^. More recently, Abdelaziz *et al.* reported that Au precursors could be reduced without reducing agents in a charged aqueous droplet reactor, which is created by the Leidenfrost phenomena, and the coating of metal oxide on three dimensional objects such as TEM grids and flexible polymeric substrates could be achieved in the same manner^23^. The interesting finding opened a new avenue for green fabrication of nanomaterials^23^,^24^.

Meanwhile, when metal nanoparticles are deposited on porous supports via a conventional incipient wetness method, especially for high metal loading, impregnation and drying procedures should be repeated several times, followed by calcination and reduction at high temperature, which usually takes a few days. As for chemical synthetic routes, reducing agents are required to reduce metal precursors and expensive equipment such as a centrifuger is needed. Thus, the development of simple and green synthesis methods for supported noble metal catalysts remains important challenges to improve their sustainability.

Here we first synthesized Pd catalysts supported on activated carbon in a green Leidenfrost droplet reactor without any reducing agents and thermal treatment at high temperature, and demonstrated that it provided good catalytic activity for formic acid decomposition at room temperature. To the best of our knowledge, there are no precedents for the preparation of supported metal catalysts in a Leidenfrost droplet reactor. Compared with conventional synthetic methods such as an incipient wetness method and chemical synthetic method, in the case of Leidenfrost droplet reactors, it takes just a few minutes to synthesize carbon supported Pd catalysts, and we do not need any reducing agents and sophisticated apparatus. The green Leidenfrost reactor requires just aqueous metal precursor solution and a hot plate to fabricate supported metal catalysts. Thus, we believe that the Leidenfrost droplet reactors can offer fresh vision for straightforward and sustainable synthesis of supported catalysts. However, in the current state, the Leidenfrost droplet reactors are considered not to be suitable for large-scale production, so further research is required to expand such synthetic method to large-scale production.

## Results

The Leidenfrost effect is a phenomenon in which a liquid droplet floats on hot surface at temperature much higher than boiling temperature of the liquid. When a liquid droplet is dripped on hot surface, vapor layer forms immediately between hot surface and the bottom of the droplet. The vapor layer levitates the liquid droplet and the levitation is maintained until the liquid is totally dried^23^,^24^. In addition, during the levitation of the liquid droplet by the Leidenfrost effect, a temperature gradient is created between the droplet and the droplet-vapor interface, and the temperature gradient induces the self-ionization of water to hydroxyl (OH^−^) and hydronium (H_3_O^+^) ions^23^,^24^. Subsequently, basic condition is established by elimination of hydronium ions to the vapor layer. Metal precursors can be reduced to neutral metal atoms under such basic condition^23^,^24^.

**Figure 1:**
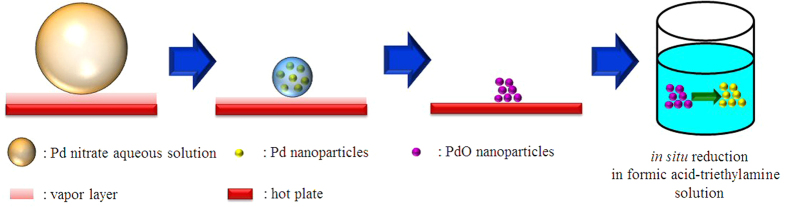
A schematic diagram for synthesis of Pd nanoparticles in the Leidenfrost droplet reactor.

**Figure 2:**
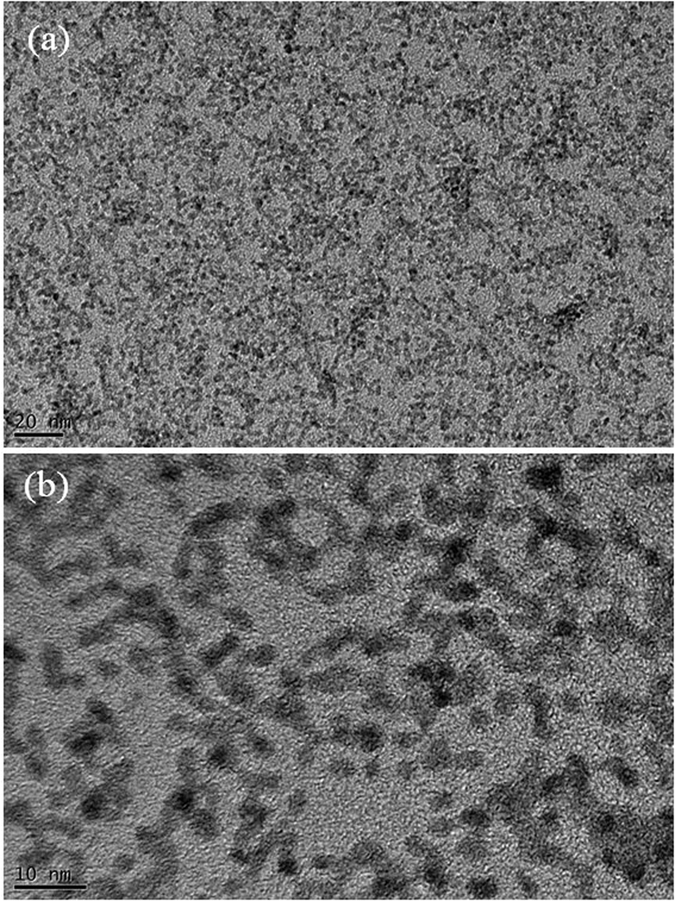
TEM images for PdO nanoparticles prepared in the Leidenfrost droplet reactor. (**a**) low magnification image. (**b**) high magnification image.

**Figure 3:**
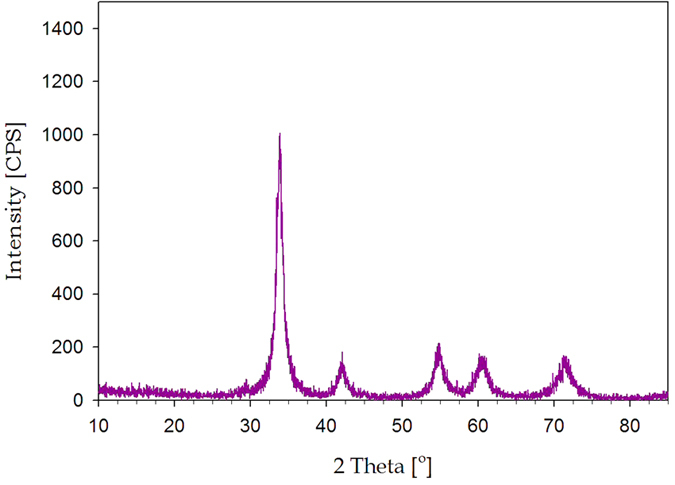
XRD patterns of PdO nanoparticles prepared in the Leidenfrost droplet reactor.

As shown in Fig. 1, Pd nanoparticles can be easily synthesized by just dripping Pd nitrate aqueous solution on a hot plate. The Pd nitrate aqueous solution droplet is levitated on a hot plate by the Leidenfrost effect. Because weakly basic condition is established by the Leidenfrost phenomena during the levitation, Pd nitrate is reduced to Pd nanoparticles. Eventually, nanoporous morphology arises from aggregation and fusion of Pd nanoparticles^24^. After complete removal of water, the as-prepared Pd nanoparticles may be oxidized to PdO on a hot plate under air atmosphere. However, when the nanoparticles are used as a catalyst for formic acid decomposition, PdO nanoparticles can be reduced again to Pd via *in situ* reduction by reactant solution containing formic acid and triethyamine. Thus Pd nanoparticles play an important role as a catalyst for formic acid decomposition. Figure 2 represents TEM images for PdO nanoparticles prepared in a Leidenfrost droplet reactor. The PdO nanoparticles with particle diameter of 2–5 nm were agglomerated and fused together to form worm-like nanoporous structure. Figure 3 shows XRD patterns for PdO nanoparticles synthesized via the Leidenfrost-effect-assisted method. The pattern of nanoparticles displayed five peaks at 33.8°, 41.9°, 54.8°, 60.3°, and 71.5°, which corresponds to PdO nanocrystals. On the basis of XRD patterns, it was revealed that the Pd nitrate precursor was successfully reduced to Pd nanoparticles in the Leidenfrost droplet reactor without any reducing agents, and subsequently oxidized to PdO under hot air atmosphere after complete elimination of water.

**Figure 4:**
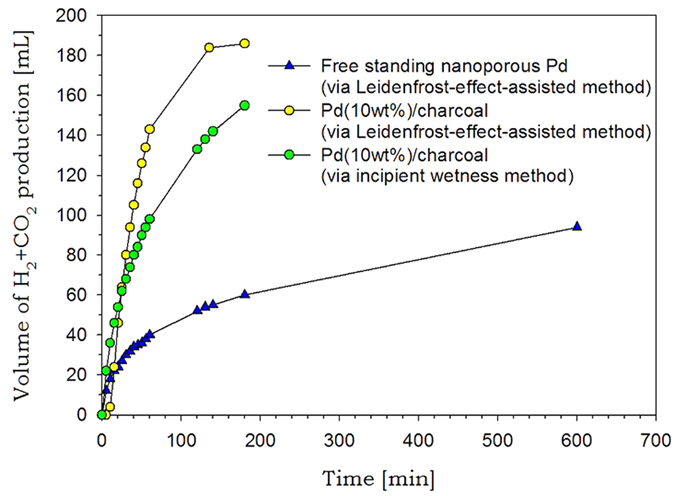
Formic acid decomposition results for free standing Pd nanoparticles, Pd(10 wt%)/charcoal prepared in the Leidenfrost droplet reactor, and Pd(10 wt%)/charcoal synthesized by incipient wetness method.

To estimate the catalytic activity of the as-prepared unsupported nanoparticles, we selected formic acid dehydrogenation reaction at room temperature. Because formic acid has non-toxicity, high hydrogen content, and availability of the existing gasoline and oil infrastructure, room-temperature hydrogen production from formic acid is a very important issue for development of low-temperature hydrogen storage and production systems^25^,^26^. We conducted formic acid decomposition tests with the unsupported nanoparticles fabricated in the Leidenfrost droplet reactor. When reactant solution containing formic acid, water, and triethylamine is mixed with the as-prepared PdO nanoparticles, PdO can be reduced again to catalytically active Pd nanoparticles by triethylamine and formic acid as a reductant (Fig. 1). As a result, 94 mL of H_2_ and CO_2_ was produced within 600 min (Fig. 4). The volumetric ratio of H_2_ to CO_2_ was 51.9:48.1, and CO was not detected (detection limit <10 ppm). The nanoparticles prepared in the Leidenfrost droplet reactor gave 2.59 mol H_2_ mol^−1^ Pd h^−1^ of a turnover frequency (TOF) at initial 30 min and 25 °C, which is superior to those of unsupported Pd catalysts reported previously (2.17 mol H_2_ mol^−1^ Pd h^−1 ^26 and 0.27 mol H_2_ mol^−1^ Pd h^−1 ^27). To the best of our knowledge, this is the first application of nanomaterials prepared in the Leidenfrost droplet reactor to heterogeneous catalysts. However, because the unsupported catalysts exhibited poor catalytic activity, nanoparticles should be deposited on porous supports to improve the catalytic activity through well dispersion of nanoparticles and strong metal-support interactions.

**Figure 5:**
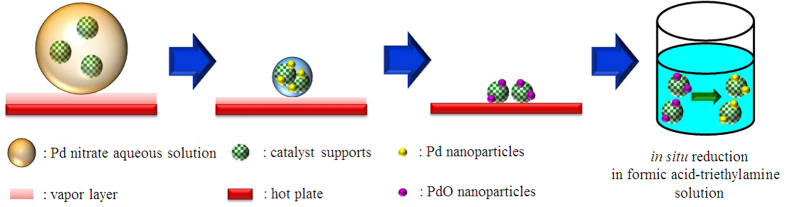
A schematic diagram for synthesis of porous-carbon-supported Pd nanoparticles in the Leidenfrost droplet reactor.

Thus, as shown in Fig. 5, we have extended the green Leidenfrost-effect-assisted method to synthesis of supported Pd catalysts. The procedures for the synthesis of supported catalyst in the green Leidenfrost droplet reactor are the same as that of unsupported Pd nanoparticles except for the addition of porous supports into the Pd precursor aqueous solution. Activated charcoal as a porous carbon support is ground into fine powder and added into Pd precursor aqueous solution, resulting in well-dispersed Pd precursor/charcoal slurry. Afterward, the slurry is dripped onto hot surface above 270 °C, leading to the reduction of Pd precursor to Pd nanoparticles during levitation of a slurry droplet on hot surface. While the droplet is boiled down, the as-prepared Pd nanoparticles are readily deposited on the activated charcoal support (supplementary movie). After complete elimination of water, the as-prepared Pd/charcoal catalysts could be oxidized to PdO/charcoal on a hot plate under air atmosphere. However, during formic acid decomposition tests, PdO nanoparticles can be readily reduced again to Pd via *in situ* reduction in formic acid-triethylamine aqueous solution.

**Figure 6:**
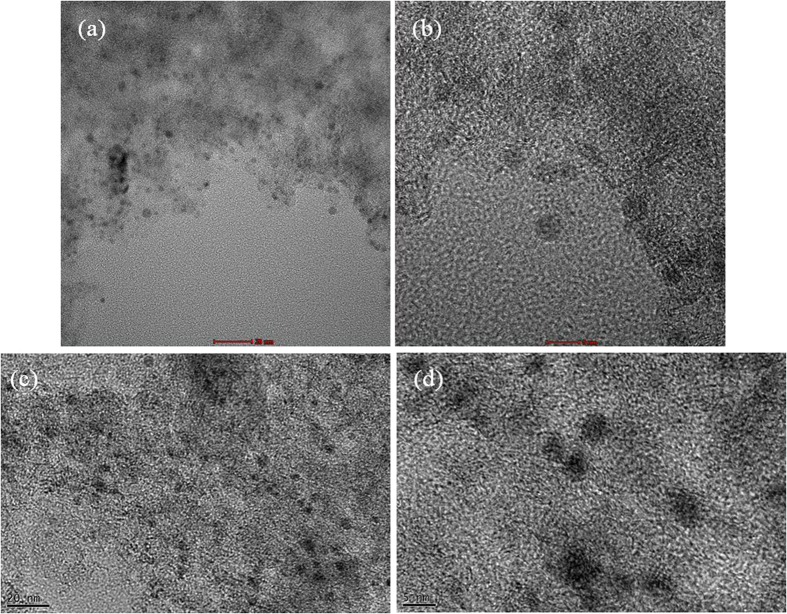
TEM images before formic acid decomposition tests for (a,b) PdO(Pd basis 10 wt%)/charcoal prepared by the Leidenfrost-effect-assisted method and (c,d) Pd(10 wt%)/charcoal prepared by incipient wetness method. (**a,c**) scale bar: 20 nm. (**b,d**) scale bar: 5 nm.

In case of the Leidenfrost-effect-assisted method without catalyst supports (Figs 1 and 2), discrete nanoparticles are not obtained. After complete removal of water, worm-like nanoporous structure is obtained because as-prepared nanoparticles are agglomerated and fused during boiling down droplets on a hot plate. However, in case of supported catalysts prepared via the Leidenfrost-effect-assisted method (Figs 5 and 6), as-prepared nanoparticles are adsorbed on support surface, which suppresses the fusion of nanoparticles. Therefore discrete nanoparticles loaded on supports can be obtained.

**Figure 7:**
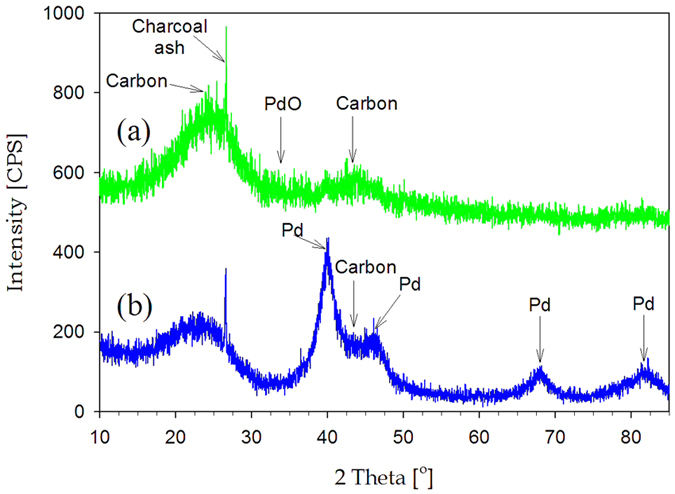
XRD patterns before formic acid decomposition tests for (a) PdO(Pd basis 10 wt%)/charcoal prepared by the Leidenfrost-effect-assisted method and (b) Pd(10 wt%)/charcoal prepared by incipient wetness method.

Figure 6a,b show TEM images for PdO(Pd basis 10 wt%)/charcoal fabricated in the Leidenfrost droplet reactor. PdO nanoparticles with particle diameter of 2.0–3.8 nm were instantly deposited and well dispersed on the activated charcoal supports. Figure 7a exhibits XRD patterns for PdO(Pd basis 10 wt%)/charcoal fabricated in the Leidenfrost droplet reactor. The pattern reveals a broad and weak PdO diffraction peak at about 33.8°, which indicates the formation of small PdO nanoparticles. In addition, the pattern displayed broad diffraction peaks at 24.6° and 43.3°, corresponding to the graphitic component of charcoal supports, and a sharp peak at 26.6° originating from the ash component of charcoal supports. What is more intriguing is that the whole process, including Pd precursor reduction and nanoparticle deposition on supports, was completed within just a few minutes and without reducing agents. In the case of conventional chemical synthetic routes, reducing agents are required to reduce metal precursors and expensive equipment such as a centrifuger is needed. However, the Leidenfrost droplet reactor does not need any reducing agents and sophisticated apparatus. Compared with conventional impregnation methods, the Leidenfrost droplet reactor is believed to be much more advanced synthetic tools due to several advantages such as short synthesis time and low carbon dioxide emission. The conventional impregnation method essentially requires high-temperature calcination and reduction procedures. However, in the case of Leidenfrost droplet reactors, such time-consuming and energy-consuming procedures are unnecessary, which allows the synthetic method to be much more straightforward and ecofriendly.

**Figure 8:**
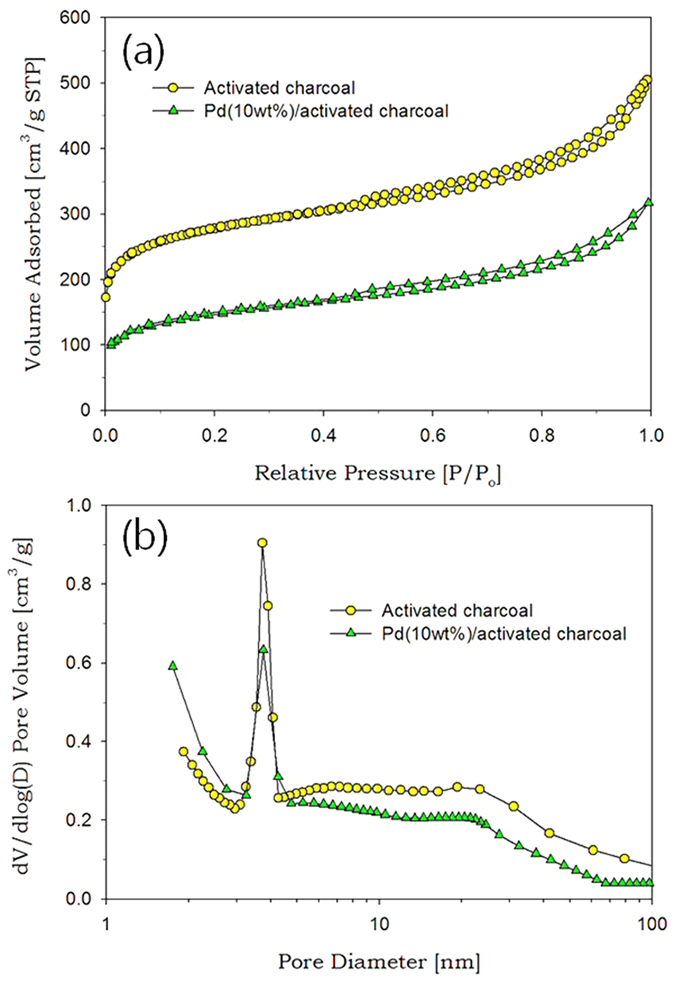
Nitrogen sorption results for activated charcoal and PdO(Pd basis 10 wt%)/charcoal prepared in the Leidenfrost droplet reactor. (**a**) isotherms. (**b**) pore size distributions.

For characterization of pore properties of the activated charcoal before and after nanoparticle deposition, nitrogen sorption tests for charcoal and PdO(Pd basis 10 wt%)/charcoal were conducted. The activated charcoal used as a catalyst support gave a typical type IV isotherm with a H3 hysteresis loop, which is commonly associated with slit-like pores derived from aggregates of plate-like particles (Fig. 8a). Its specific surface area and pore volume were 1030 m^2^/g and 0.78 cm^3^/g, respectively. In addition, charcoal provided trimodal pore size distribution, being composed of micropores below 2 nm, small mesopores centered at 3.8 nm, and large mesopores above 10 nm. After PdO deposition on the activated charcoal by the Leidenfrost-effect-assisted method, specific surface area and pore volume of the charcoal significantly decreased to 516 m^2^/g and 0.49 cm^3^/g. As shown in Fig. 8b, the mesopore volume substantially decreased due to PdO nanoparticle deposition. It was revealed that along with TEM images, the nitrogen sorption results also demonstrated the successful deposition of nanoparticles on charcoal supports via the Leidenfrost-effect-assisted method. When PdO/charcoal is used as a catalyst for formic acid decomposition, the PdO nanoparticles supported on charcoal can be readily reduced again to Pd via *in situ* reduction during formic acid decomposition reaction.

For the comparison of the Leidenfrost droplet reactor with conventional methods, we also prepared Pd (10 wt%)/charcoal by means of incipient wetness. As shown in Fig. 6c,d, Pd nanoparticles with particle diameter of 2.8–5.2 nm were deposited on the activated charcoal supports. Figure 7b shows XRD patterns for Pd(10 wt%)/charcoal synthesized by incipient wetness method. The pattern shows broad Pd diffraction peaks at 40.1°, 68.1°, and 81.9°. Compared to the Pd diffraction peak in Fig. 7b, the PdO diffraction peak in Fig. 7a is much broader. On the basis of TEM images (Fig. 6) and XRD patterns (Fig. 7), it was revealed that the nanoparticle size for the catalysts prepared by incipient wetness method was larger in comparison with the catalysts synthesized in the Leidenfrost droplet reactor. Even if the Leidenfrost droplet reactor is a much simpler and more ecofriendly synthetic tool and requires much shorter synthesis time than the conventional impregnation method, the catalysts prepared in the Leidenfrost droplet reactor provided smaller particle size than incipient wetness method.

Using the as-prepared catalysts, we conducted formic acid decomposition tests without stirring in a closed stainless steel reactor connected with a gas burette at 25 °C. Figure 4 shows plots of produced gas (H_2_ and CO_2_) volume versus reaction time for the catalysts prepared by the Leidenfrost-effect-assisted method and incipient wetness. In the case of catalysts prepared by incipient wetness, 155 mL of H_2_ and CO_2_ was produced within 180 min, and turnover frequency (TOF) at initial 30 min and 25 °C was 58.7 mol H_2_ mol^−1^ Pd h^−1^. In contrast, the catalysts synthesized in the Leidenfrost droplet reactor gave 69.1 mol H_2_ mol^−1^ Pd h^−1^ of turnover frequency (TOF) at initial 30 min and 25 °C, and 184 mL of H_2_ and CO_2_ was produced within 180 min, which is higher than that of the catalysts prepared by incipient wetness. In general, smaller metal nanoparticles lead to higher catalytic activity because more active sites are available to reactants. Thus it can be concluded that the catalysts prepared in the Leidenfrost droplet reactor showed better catalytic activity than those synthesized by the incipient wetness method, and smaller nanoparticles deposited on charcoal may be responsible for better performance.

However, in the range of reaction time between 0 min and 20 min, the generated gas volume for the catalysts synthesized in the Leidenfrost droplet reactor was lower than that for the catalysts synthesized by incipient wetness (Fig. 4). To elucidate such a phenomenon and confirm the *in situ* reduction of PdO/charcoal into Pd/charcoal during formic acid decomposition tests, we carried out XPS and XRD analyses before and after formic acid decomposition tests for the catalysts synthesized in the Leidenfrost droplet reactor.

**Figure 9:**
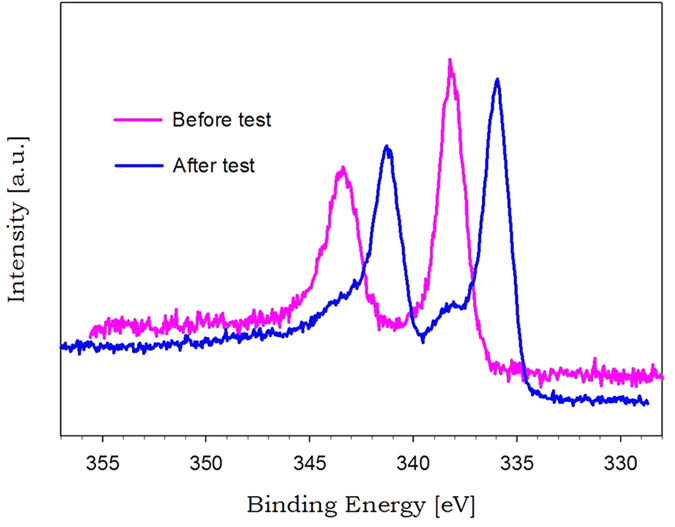
XPS spectra before and after formic acid decomposition tests for the catalysts prepared by the Leidenfrost-effect-assisted method.

**Figure 10:**
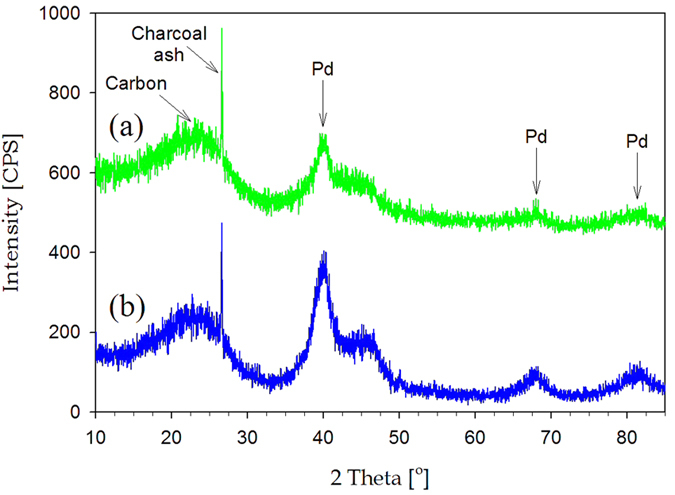
XRD patterns after formic acid decomposition tests for Pd(10 wt%)/charcoal catalysts. (**a**) Pd(10 wt%)/charcoal prepared in the Leidenfrost droplet reactor. (**b**) Pd(10 wt%)/charcoal prepared by incipient wetness method.

Figure 9 exhibits XPS spectra before and after formic acid decomposition test for the catalysts synthesized in the Leidenfrost droplet reactor. The catalysts before formic acid decomposition tests have two intense peaks at 338.3 eV and 343.7 eV, corresponding to 3d_5/2_ and 3d_3/2_ components for PdO. However, after the tests, the catalysts provided peaks at 336.0 eV and 341.4 eV, being ascribed to 3d_5/2_ and 3d_3/2_ components for the zero-valence metallic state of Pd nanoparticles with high electron density^25^. The shift toward low binding energy indicates PdO reduction to Pd during formic acid decomposition tests. Figure 10a shows XRD patterns after formic acid decomposition tests for the catalysts synthesized in the Leidenfrost droplet reactor. The pattern provided broad Pd diffraction peaks at 40.1°, 68.1°, and 81.9°, and displayed diffraction peaks at 24.6°, 26.6°, and 43.3° originating from the charcoal supports. Comparing Fig. 10a with Fig. 7a, it was confirmed that PdO/charcoal prepared in the Leidenfrost droplet reactor was easily reduced to Pd/charcoal catalysts during formic acid decomposition tests. On the basis of XPS (Fig. 9) and XRD (Figs 7a and 10a) results before and after formic acid decomposition tests, it was revealed that the nanocrystalline structure of the as-prepared catalysts via the Leidenfrost-effect-assisted method was PdO, and PdO was reduced again to Pd nanoparticles during the formic acid decomposition tests. The triethylamine, formic acid, or produced hydrogen might be attributed to the *in situ* reduction of PdO to Pd nanoparticles, and the range of reaction time between 0 min and 20 min showing lower catalytic performance is considered to be the period of the *in situ* reduction.

To investigate the correlation of Pd nanoparticle size with better performance of the catalysts prepared in the Leidenfrost droplet reactor, we conducted XRD and chemisorption analyses after formic acid decomposition tests for the Pd(10 wt%)/charcoal catalysts prepared by the Leidenfrost-effect-assisted method and incipient wetness method. As shown in XRD patterns (Fig. 10), the Pd/charcoal catalysts prepared in the Leidenfrost droplet reactor gave broader Pd diffraction peaks in comparison with that prepared by the incipient wetness method, indicating that Pd nanoparticle size for the catalysts prepared in the Leidenfrost droplet reactor is smaller than that for the catalysts prepared by the incipient wetness method. In addition, chemisorption analyses provided Pd metal dispersion and crystalline size for the catalysts (Table 1). As a result, Pd metal dispersion and Pd crystalline size for Pd/charcoal catalysts prepared in the Leidenfrost droplet reactor were 42.1% and 2.7 nm, and those for the catalysts prepared by the incipient wetness method were 28.0% and 4.0 nm, respectively. Thus, on the basis of XRD (Fig. 10) and chemisorption results (Table 1), it can be demonstrated that better performance of Pd/charcoal catalysts prepared in the Leidenfrost droplet reactor was attributed to their smaller Pd particle size and much higher Pd dispersion than the catalysts prepared by the incipient wetness method.

## Discussion

We first synthesized Pd nanoparticles supported on activated charcoal in the Leidenfrost droplet reactor. Compared to a conventional incipient wetness method, charcoal-supported Pd nanoparticles can be fabricated in a much shorter time, and the Leidenfrost droplet reactor is more ecofriendly and requires nothing except for Pd precursor aqueous solution, charcoal supports, and a hot plate. Despite the shorter synthesis time, charcoal-supported Pd nanoparticles provided smaller particle size than those prepared by means of incipient wetness. In addition, we first utilized the charcoal-supported Pd nanoparticles prepared in the Leidenfrost droplet reactor as a catalyst for formic acid decomposition reaction. As a result, TOF of Pd/charcoal prepared in the Leidenfrost droplet reactor increased by 17.7% in comparison with that prepared by incipient wetness. Such an improvement in catalytic activity is attributed to smaller Pd nanoparticle size achieved by the Leidenfrost droplet reactor. The extension of the Leidenfrost-effect-assisted method to different supported metal catalysts is underway. Such a straightforward synthetic pathway is believed to be helpful in developing faster methods for catalyst screening.

## Methods

### Synthesis of Pd Nanoparticles in the Leidenfrost Droplet Reactors

A droplet of Pd(NO_3_)_2_ aqueous solution (10 wt% Pd basis, PMRESEARCH Co.) was dripped on a hot plate heated above 270 °C. While the droplet levitated on the hot plate, the droplet was dried, and its color changed from orange to black. After drying of the droplet, a black flake of Pd nanoparticles remained on the hot plate^24^.

### Synthesis of Pd/Charcoal Catalysts in the Leidenfrost Droplet Reactors

0.18 g of activated charcoal (Aldrich) was finely ground and added into 1.2 mL of distilled water, followed by the addition of 0.2 g of Pd(NO_3_)_2_ aqueous stock solution (10 wt% Pd basis) into the mixture. The final mixture solution was sonicated for 3 min to make charcoal particles disperse well. A droplet of the final mixture was dripped on a hot plate above 270 °C, resulting in the levitation of the droplet on a hot plate. After boiling it down during the levitation, catalysts were instantly synthesized. Such a process was repeated a few times until the prepared solution was completely consumed. For comparison, we also prepared Pd(10 wt%)/charcoal catalysts by means of a conventional incipient wetness method. In a typical synthesis, 0.2 g of Pd(NO_3_)_2_ aqueous stock solution (10 wt% Pd basis) was diluted with 1.2 mL of distilled water to prepare the Pd nitrate solution for incipient wetness. Subsequently, 0.18 g of activated charcoal was impregnated with the as-prepared Pd nitrate aqueous solution, and then Pd-nitrate-impregnated charcoal samples were dried at 100 °C for 1 h. Such impregnation and drying process was repeated several times until the as-prepared Pd nitrate solution was totally consumed. Afterward the Pd-nitrate-impregnated sample was reduced at 350 °C for 2 h under hydrogen atmosphere, resulting in Pd(10 wt%)/charcoal catalysts.

### Catalytic Decomposition of Formic Acid at Room Temperature

0.055 g of Pd(10 wt%)/charcoal catalysts was sealed in a 100 mL Teflon-lined stainless steel reactor, followed by a nitrogen purge for 30 min. After the nitrogen purge, the outlet of the reactor was connected to a gas burette system filled with water. Subsequently, a mixture of 10 mL of distilled water, 0.19 mL of formic acid (95%, Aldrich), and 0.14 mL of triethylamine (99%, Aldrich) was injected into the reactor through a rubber septum. The volume of gas generated at 25 °C was measured by the gas burette system.

### Characterization

The pore properties of activated charcoal and Pd/charcoal catalysts were taken by nitrogen sorption tests with a Micromeritics ASAP 2420 instrument. Degassing of samples was conducted at 200 °C for 5 h. H_2_ chemisorption of the catalysts after formic acid decomposition tests was carried out by using Micromeritics ASAP 2020 instrument. Transmission electron microscopy (TEM) analyses were conducted by using a FEI/TECNAI G2 instrument. X-ray photoelectron spectroscopy (XPS) was performed using a Kratos 165XP spectrometer. X-ray diffraction (XRD) patterns were collected on a Rigaku D/MAX-2200 V instrument operated at 1.6 kW.

## Additional Information

**How to cite this article**: Lee, D.-W. *et al.* Reducing-Agent-Free Instant Synthesis of Carbon-Supported Pd Catalysts in a Green Leidenfrost Droplet Reactor and Catalytic Activity in Formic Acid Dehydrogenation. *Sci. Rep.* **6**, 26474; doi: 10.1038/srep26474 (2016).

## Supplementary Material

Supplementary Information

supplementary movie

## Figures and Tables

**Table 1:** Chemisorption results for Pd(10 wt%)/charcoal catalysts after the formic acid decomposition test.

Sample	Pd metal dispersion [%]	Pd nanocrystalline size [nm]
Pd(10 wt%)/charcoal prepared in the Leidenfrost droplet reactor	42.1	2.7
Pd(10 wt%)/charcoal prepared by incipient wetness method	28.0	4.0
