# Supplementary material for: Reducing-Agent-Free Instant Synthesis of Carbon-Supported Pd Catalysts in a Green Leidenfrost Droplet Reactor and Catalytic Activity in Formic Acid Dehydrogenation
Source: Sci Rep. 2016 May 20;6:26474. doi: 10.1038/srep26474 (PMC4873793; doi:10.1038/srep26474)
Supplement: Supplementary Information [file srep26474-s1.doc]

**Supplementary Information**

**Reducing-Agent-Free Instant Synthesis of Carbon-Supported Pd Catalysts in a Green Leidenfrost Droplet Reactor and Catalytic Activity in Formic Acid Dehydrogenation**

**Dong-Wook Lee^1,*^, Min-Ho Jin^1^, Young-Joo Lee^2^, Ju-Hyoung Park^2^, Chun-Boo Lee^1^, and Jong-Soo Park^1^**

**^1^** Advanced Materials and Devices Laboratory, Korea Institute of Energy Research (KIER),

*152 Gajeongro, Yuseong, Daejeon 305-343, Republic of Korea,*

**^2^** Clean Fuel Laboratory, Korea Institute of Energy Research (KIER),

*152 Gajeongro, Yuseong, Daejeon 305-343, Republic of Korea*

** corresponding author: dwlee99@kier.re.kr*

**- Supplementary movie legend**

Supplementary Movie. Video for synthesis of Pd(10wt%)/charcoal catalysts by the Leidenfrost-effect-assisted method.
